# 

**Published:** 1903-01-15

**Authors:** 


					﻿THE
DENTAL REGISTER
EDITED BY
NELVILLE S. HOFF, D.D.S.,
ANN ARBOR, MICH.
Voi. LVII. JANUARY 15, 1903.	No. 1.
PRICE, ONE DOLLAR A YEAR IN ADVANCE,
PUBLISHED MONTHLY BY
SAMUEL· A. CROCKER & CO.,
THE OHIO DENTAL AND SURGICAL DEPOT.
to 30 W. "tin St., Cincinnati, O.
Entered at the Postoffice at Cincinnati, 0., as second-class mail matter.
				

